# Therapeutics

**Published:** 1876-05

**Authors:** 


### IV. Therapeutics.

1. *Whooping Cough Treated by Complete Etherization.* Dr. S. D. Powell. (*Ex.*)

At the last meeting of the Neurological Society, Dr. P. advocated the above cure for pertussis. The etherization must be continued forty or fifty minutes. He related about six cases in which this procedure had proved successful, though in one or two cases the anæsthetic had to be given a second time. He hit upon this method of treatment accidentally from etherizing a child who had pertussis at the time, for the reduction of a fracture.

2. *Tooth Ache.* C. A. Guild. (*Clinic.*)

Equal parts of collodion and carbolic acid, form a stiff jelly, which may be applied with a pine stick to the cavity of an aching tooth. The pain will be relieved almost instantly if it depend on an exposed nerve.

3. *Salicylic Acid for Articular Rheumatism.* Dr. Stricker. (*Berl. Klin. Wochenschr.; Allg. Med. Central. Ztg.*, 19, 1876.)

Since Dr. Stricker first advocated the internal exhibition of the salicycic acid for rheumatism, he has received a great many communications from professional friends confirming the good effect of the remedy on the disease. The success depends, however, very much on the purity of the acid. He puts his experience down in the following sentences:

1. The salicylic acid seems to be a prompt and reliable remedy for fresh cases of genuine articular rheumatism.

2. It is not injurious to the human organism if given in hourly doses of 7 to 15 grains (0.5 to 1.0 gramme.)

3. Such doses can be continued longer in young and robust persons than in old and feeble ones.

4. It produces the signs of intoxication sooner with the latter than with the former.

5. This intoxication shows various grades; tinnitus aurium, dullness of hearing, and perspiration, are the ordinary signs, and yet they do not contra-indicate the further use of the acid.

6. To prevent a recidive the acid should be continued in small doses (20 to 40 grains pro die, for at least one week longer.

7. In chronic cases of articular rheumatism its efficiency is doubtful, and in so-called gonorrhoic rheumatism it is very improbable.

4. *Opium and its Antidote.* Dr. J. H. Stearns. (*Detroit Review of Medicine*, April, 1876)

It is admitted that opium, in its several forms, is the great antidote for pain, and in prescribing it the dose should be regulated by the extent and severity of the pain, modified by the fact as to whether the patient's system has recently been accustomed to its use.

The custom of giving opium or morphine for mere sleeplessness can not be too highly reprehended, or to give it where the pains are fugitive or uncertain of duration, because should the pain disappear, the full force of the drug would be expended on the brain, with perhaps dangerous or fatal results.

As to antidotes for an over-dose of opium, the brain of the profession has been exercised to an alarming extent. I believe that atropia is now the fashionable thing to exhibit hypodermically.

Realizing that opium is the antidote for pain, and that otherwise deadly doses are carried off with impunity where the pain is excessive, we have only to reverse the proposition, and the question is solved, for we have pain as the antidote for opium. Finding the patient laboring under an over-dose of any opiate, proceed at once to inflict upon him a pain that will be persistent and continued at your pleasure. Remembering that the most excruciating torture is experienced by pressure upon terminal branches of the nerves, the most convenient way will be to apply pressure upon the ends of the thumbs and fingers. This can be done by using a small hand vice, or even the ordinary spring clothes-pins will answer every purpose, for it is astonishing to experience the amount of pain one of these little instruments will inflict with its persistent grip.

The inquisition found the thumb-screw the most effectual machine for torture that could be advised. Ephemeral and transient pains will be of little good; it is the steady and continued agony that accomplishes the work.

Accounts of cases treated on the theory indicated would be of little use, unless it commends itself to the judgment, and this paper is respectfully submitted to the readers of the *Review*, trusting that it will receive a careful examination, in the light of previous knowledge and experience with this powerful drug.

5. *Grindelia Robusta—Therapeutic Action and Chemical Analysis.* C. J. Rademaker, M.D. (*Louisville Med. News.*)

I have used this medicine in several cases of chronic pneumonia, bronchitis, and in asthmatic troubles, whether depending on cardiac lesions or structural disease of the lungs or bronchial tubes, and my patients were invariably benefited by it to some extent.

Mrs. F., aged 48, had chronic pneumonia of about two years' duration, with considerable purulent expectoration. Physical examination of the chest revealed flatness of the entire left lung. She suffered with considerable dyspnœa, and had used all the expectorants, oils, anodynes, tonics, alteratives and alkalies in existence, with little benefit. She was given one teaspoonful of the fluid extract of grindelia robusta every two or three hours, or more frequently when her cough was troublesome. Under this treatment the cough was less severe, the expectoration diminished, and her appetite and general health improved so that she is better now than she has been for a year.

CASE II.—Mrs. R. had pneumonia about eighteen months ago. She has flatness of the right lung and partial consolidation at the base of the left remaining. I treated her in the acute stage of pneumonia and am treating her now. She had many remedies given her without any benefit. I placed her under the fluid extract of grindelia robusta, giving her a teaspoonful every two hours. She was put on this treatment about three weeks ago, and as long as she has been under it she sleeps better, has less cough and dyspnœa, and is in all respects much better than she has been for nine months.

CASE III.—Has cardiac enlargement, with valvular lesions and chronic bronchitis. This patient formerly derived much benefit from the fluid extract of gelseminum and extract belladonna, but lately could not be so relieved. He was placed under the grindelia. In this case, as in the former, the patien t was relieved in a few days. His heart trouble o course remained the same, but his bronchitis was seemingly entirely cured.

I have used this medicine also in acute attacks of pneumonia and bron chitis, but derived very little benefit from it. I think it is indicated in the chronic stages only.

*Chemical Analysis.*—One pound of herba grindelia robusta was ex hausted with a hydro-alcoholic menstruum by percolation, and the percolate reduced by evaporation to about eight fluid ounces. The remaining liquid was treated with caustic alkali in excess, and then agitated with severa times its volume of ether, the ether separated and allowed to evaporate The oleoresinous mass thus obtained had the physical appearance of gum tolu, in odor resembling gum turpentine. By dry distillation it yielded a volatile oil, the odor of which resembled oil of turpentine. Part of the oleoresin thus obtained was treated with dilute nitric acid and filtered, the filtrate decomposed with caustic potash and agitated with ether, the ether separated and allowed to evaporate spontaneously. A solution of the mass thus obtained had a distinct alkaline reaction, and under the microscope showed well formed prismatic crystals.

The alkaline fluid from which the base and oleoresin had been extracted by means of ether, was now treated with dilute sulphuric acid, in orde to obtain the organic acid present. Sulphuric acid being added until the solution had a distinct acid reaction, the acid solution was then agitated with several times its volume of ether, the ether separated and allowed to evaporate. The residue was then exhausted with distilled water, in order to separate the acid from any resinous matter that had been extracted with it by ether. The aqueous solution thus obtained had a distinct acid reaction, completely neutralizing alkalies and forming salts. The solution of these salts had a yellow color. Under the microscope the acid showed well formed accicular crystals.

Time not permitting me to make a further investigation of the acid and base of this drug at present, at some future time I will make a further chemical examination, and also upon what part of the drug its physiological and active properties depend.

